# Epidemiological and microbiological characterization of *Candidozyma auris* (*Candida auris*) isolates from a tertiary hospital in Cairo, Egypt: an 18-month study

**DOI:** 10.1038/s41598-025-15302-3

**Published:** 2025-09-12

**Authors:** Fatma Ahmed, Iman M. El-Kholy, Dalia A. M. Abdou, Adel A. El-Mehalawy, Nadia A. Elkady

**Affiliations:** 1 Microbiology Department, Faculty of Science, Ain Shams University, Cairo, Egypt; 2 Clinical Pathology Department, Ain Shams University Specialized Hospital, Ain Shams University, Cairo, Egypt

**Keywords:** *Candida auris*, Epidemiology, Multidrug resistance, Virulence, Biofilm, Hemolysis, Microbiology, Medical research

## Abstract

*Candidozyma auris* (formerly *Candida auris*) has emerged as a significant multidrug-resistant pathogen. Among 140 antifungal-resistant *Candida* spp. isolates, 120 were identified as *C. auris* using chromogenic agar, VITEK 2, and MALDI-TOF. Most cases (60%) were males, and 59% were aged ≥ 60 years. Bloodstream infections were predominant (60.8%), followed by respiratory (20.8%), urinary tract (15%), and other sites. Liver transplantation (35.8%) was the most common underlying risk factor, followed by post-COVID-19 (30.8%) and cancer (25%). All isolates exhibited thermotolerance, halotolerance, anaerobic blood hemolysis, and biofilm formation. Significant association was observed between virulence enzymes activity and both specimen source and clinical conditions (*P* < 0.001), with strong activity linked to blood isolates, liver transplant, post-COVID-19, and lung cancer. Among antifungal agents, voriconazole (MIC_50_/_90_: 0.125/0.5 µg/mL), posaconazole (0.03/0.125 µg/mL), and amphotericin B (0.5/1 µg/mL) showed consistently low MICs. Caspofungin (0.25/1 µg/mL) and micafungin (0.125/0.5 µg/mL) demonstrated favorable activity, with resistance rates of 3.3% and 4.2%, respectively. Fluconazole resistance was observed among all isolates (MIC_50_/_90_: 32/32 µg/mL). Itraconazole and ketoconazole showed elevated MICs (MIC_50_/_90_: 1/4 and 16/64 µg/mL, respectively) and was inactive against all isolates. Flucytosine (MIC_50_/_90_: 64/128 µg/mL) was inactive against 96.7%. Agreement between VITEK 2 and CLSI BMD was strong for fluconazole, voriconazole, amphotericin B, caspofungin, and micafungin (Cohen’s kappa ≥ 0.8), but poor for flucytosine (0.35), indicating limited reliability. This report provides crucial local data on antifungal susceptibility and virulence traits of *C. auris*, supporting infection control and treatment strategies in Egypt.

## Introduction

*Candidozyma auris* (formerly *Candida auris*) is a relatively new fungal pathogen. Its emergence on the global healthcare scene has been swift and concerning, raising questions about its origin and the factors contributing to its rapid spread. Known for its ability to cause severe, invasive infections, especially in hospital and healthcare settings, *C. auris* poses unique challenges due to its resistance to antifungal drugs, ability to survive on surfaces for extended periods, and its remarkable ability to colonize and infect individuals in diverse healthcare settings^1^.

The initial reports of *C. auris* infections appeared in Japan and India in 2009 and 2011, respectively, among patients with severe underlying health conditions^2,3^. Interestingly, retrospective studies expect that *C. auris* infections likely began several years before their official identification. A previous study revised some *C. haemulonii* isolates collected before 2009 from Asia, Europe, Latin America, and North America using matrix-assisted laser desorption/ionization time-of-flight mass spectrometry (MALDI-TOF MS), confirmed that few cases of *C. auris* occurred before its initial report^4^. These findings indicated that *C. auris* had been circulating undetected for some time, raising questions about how widespread it was by the time of its discovery.

The name *C. auris* originates from the Latin word *auris*, meaning “ear,” referring to its initial isolation from a case of otitis media^2^. This nomenclature initially suggested that *C. auris* causes only localized infections. However, later investigations revealed a more concerning pattern. *C. auris* can display a wide range of manifestations, including candidemia, urogenital tract, respiratory tract, central nervous system, and wound infections, added to otitis media, and skin colonization^5^.

*Candidozyma auris* exhibits intrinsic resilience, allowing it to persist in clinical settings, rapidly colonize patients’ skin, and facilitate efficient transmission within healthcare environments, contributing to severe and prolonged outbreaks^5^. The rapid spread of *C. auris* and its ability to cause persistent infections in hospitals have made it a significant public health threat. In 2019, the U.S. Centers for Disease Control and Prevention (CDC) identified *C. auris* as the sole fungal pathogen among the five most urgent threats in its *Antibiotic Resistance Threats Report*^6^. More recently, the World Health Organization (WHO) ranked *C. auris* second in the critical category of its *Fungal Pathogens Priority List*^7^. This was not only due to its multidrug resistance but also because of the high mortality rates associated with bloodstream infections, especially in vulnerable patients. Some studies reported mortality rates of 30–50%, although these figures are complicated by the fact that many patients infected with *C. auris* often have other severe underlying conditions^8^.

Recent findings indicate that *C. auris*, like *Candida albicans*, produces several well-established virulence factors, such as secreted aspartyl proteases (Saps) and lipases, which aid in tissue invasion and degradation^9^. In general, traits such as adherence, biofilm formation, hemolysin activity, and the production of phospholipases and proteinases have been implicated in the pathogenicity of *Candida* species, contributing to invasive fungal infections in hosts^10,11^. Biofilm formation is a well-known survival strategy in various *Candida* species, including *C. auris*, enabling cells to resist antifungal agents at concentrations significantly higher than those needed to eliminate free-floating (planktonic) cells^12^.

*Candidozyma auris* also exhibits an inherent resistance to frontline antifungal agents, with many strains showing resistance to multiple antifungal classes, including azoles, echinocandins, and polyenes, which limits treatment options^13^. Pan-antifungal-resistant strains have recently been reported^13,14^. Although pan-resistant strains remain relatively rare, their recent emergence is a significant cause for concern^13^. The growing threat of antifungal resistance in *C. auris* highlights the urgent need for ongoing surveillance, stringent infection control strategies, and the development of new therapeutic agents to effectively manage *C. auris* infections^13,15^.

*Candidozyma auris* emerged independently and simultaneously in four different regions worldwide, a conclusion supported by whole genome sequencing data^16^. Geographically, isolates of *C. auris* are categorized into six primary clades, each differing by thousands of single-nucleotide polymorphisms: clade I (Southern Asia), clade II (Eastern Asia), clade III (Africa), clade IV (South America), clade V (Iran), and clade VI (Indo-Malaysian)^16,17^. Over time, the epidemiology of invasive *C. auris* infections has significantly changed, shifting from sporadic cases to more frequent hospital-associated outbreaks involving a growing number of patients^18,19^. *Candidozyma auris* has now been isolated in over 50 countries across six continents since its discovery^7,16,20^.

Unfortunately, there is currently a lack of comprehensive studies regarding the status of *C. auris* in Egypt. This study aims to investigate the epidemiological characteristics, antifungal susceptibility testing patterns, and virulence factors of *C. auris*, shedding light on its status in Egypt and contributing data to global databases concerning this emerging fungal pathogen.

## Materials and methods

**Fig. 1:**
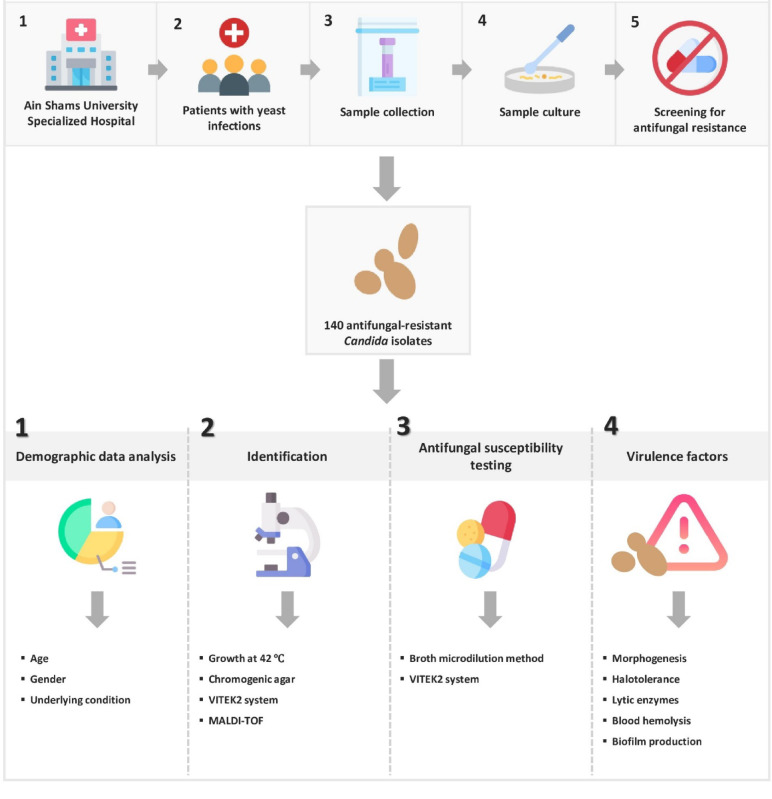
Workflow of the study (www.flaticon.com).

*Candidozyma auris* (formerly *Candida auris*) isolates were obtained from clinical specimens collected over a period of 18 months at a large tertiary care facility in Egypt. Their distribution and relative prevalence among antifungal-resistant *Candida* species were analyzed. In addition, antifungal susceptibility profiles and key virulence factors of *C. auris* were evaluated (Fig. 1).

### Sample collection

A total of 140 antifungal-resistant *Candida* isolates were recovered from different clinical specimens including urine, blood, sputum, and various body fluids over the period from May 2022 to November 2023. Clinical specimens were collected from outpatients and inpatients of all ages, genders, and health conditions at Ain Shams University Specialized Hospital (ASUSH). ASUSH is a large tertiary healthcare complex in Cairo, Egypt. It serves a diverse patient population across various specialties including internal medicine, surgery, pediatrics, gynecology, geriatrics, and emergency medicine. The hospital has a total of 666 beds, including ICU beds, and receives an average of 2400 patient admissions per month^21^.

The samples were collected as a part of routine diagnostic assessment for fungal infections. To differentiate between colonization and true infection, the criteria outlined by the European Organization for Research and Treatment of Cancer/Mycoses Study Group (EORTC/MSG) were applied^22^. Proven invasive candidiasis was defined by isolating *C. auris* from sterile body fluids or tissue. For normally non-sterile sites, diagnosis required clinical signs supported with radiological abnormalities or histopathological proof of fungal invasion. Colonization was defined as the isolation of *C. auris* from non-sterile sites in the absence of clinical or radiographic signs of infection.

Deidentified demographic variables including age and sex were collected and analyzed descriptively. The study was approved by Ain Shams University Specialized Hospital. All methods were performed in accordance with the relevant guidelines and regulations. Informed consent was obtained from all participants and/or their legal guardians.

### Identification of clinical isolates

Isolates were identified according to the U.S. Centers for Disease Control and Prevention (CDC) guidelines for *C. auris* identification. They were initially screened for their ability to grow at 42 ℃ on Sabouraud’s dextrose agar (SDA), potato dextrose agar (PDA), and brain heart infusion agar (BHI) for 48 h^23^. The isolates were then identified by selective chromogenic agar media (Brilliance™ *Candida* Agar), Vitek 2® Compact (version 8.01, bioMérieux, Marcy-L’Etoile, France)^23,24^. The identification was further confirmed by proteomic profiling using matrix-assisted laser desorption/ionization time-of-flight mass spectrometry (MALDI-TOF MS) (LT2 plus, SAI, UK) using updated reference database BactoScreen-ID version 2.4 including *C. auris* spectra^25,26^.

### Antifungal susceptibility testing

All the isolates were tested for antifungal susceptibility to fluconazole (Pfizer Inc., New York, NY, USA), itraconazole (Sigma-Aldrich, St. Louis, MO, USA), voriconazole (Pfizer Inc., New York, NY, USA), ketoconazole (Sigma-Aldrich, St. Louis, MO, USA), posaconazole (Merck & Co., Inc., Kenilworth, NJ, USA), amphotericin B (Sigma-Aldrich, St. Louis, MO, USA), flucytosine (Sigma-Aldrich, St. Louis, MO, USA), caspofungin (Merck & Co., Inc., Kenilworth, NJ, USA), and micafungin (Astellas Pharma Inc., Tokyo, Japan). Antifungal susceptibility testing was initially performed using the broth microdilution (BMD) method according to the Clinical and Laboratory Standards Institute (CLSI) M27-A3 protocol^27^, and the results were further confirmed using the VITEK 2 system (bioMérieux, France). As CLSI and the European Committee on Antimicrobial Susceptibility Testing (EUCAST) have not yet defined antifungal breakpoints for *C. auris*, tentative breakpoints proposed by the CDC were used^28^. These include fluconazole at ≥ 32 µg/mL, amphotericin B at ≥ 2 µg/mL, micafungin at ≥ 4 µg/mL, and caspofungin at ≥ 2 µg/mL.

Previously reported epidemiological cutoff values determined by the derivatization method (dECOFFs) for *C. auris* were applied to distinguish minimum inhibitory concentration (MIC) values that exceeded the wild-type distribution for voriconazole (1 µg/mL), posaconazole (0.125 µg/mL), and itraconazole (0.25 µg/mL)^29,30^. MIC values of flucytosine were compared to the CLSI breakpoint from other *Candida* species (≥ 32 µg/mL)^27,31^. However, no clinical breakpoints or epidemiological cutoff values (ECVs) have been established by CLSI or EUCAST for ketoconazole against *Candida* species. Therefore, MIC values were descriptively reported without categorical interpretation. For comparative purposes, MIC distributions were analyzed, and values ≥ 1 µg/mL were cautiously considered as potentially indicative of reduced susceptibility, based on previous reports on related *Candida* species^32,33^.

Essential agreement (EA) between VITEK 2 and CLSI BMD method was also tested. It was defined as a difference of no more than two MIC dilutions. Categorical agreement (CA) was obtained when the MIC result fell within the same interpretive categories according to suggested breakpoints and dECOFF values^27–33^. Interrater reliability testing was then used to assess EA and CA between VITEK 2 and CLSI BMD methods. Cohen’s kappa measures the agreement between EA and CA on a categorical outcome, accounting for the possibility of random agreement. A higher kappa value indicates better agreement beyond what would be expected by chance. Cohen suggested interpreting kappa values as follows: ≤ 0 as no agreement, 0.01–0.20 as slight, 0.21–0.40 as fair, 0.41–0.60 as moderate, 0.61–0.80 as substantial, and 0.81–1.00 as almost perfect. In cases of discrepant results between the two methods, testing was repeated three times for the isolate in question. The result that was consistent across the three repetitions was considered final.

### Testing virulence factors of *C. auris*

All recovered *C. auris* strains were evaluated for morphogenesis, tolerance to osmotic stress, hemolytic activities, proteolytic activities, lipolytic activities, esterase production, and biofilm formation.

#### Morphogenesis and chlamydospore production

An isolated colony of *C. auris* from a purified culture was evenly inoculated on a cornmeal agar plate. A coverslip was placed over the inoculated area to create microaerophilic conditions. The plates were incubated at 30 °C for 5 days in the dark. After incubation, the plates were examined for evidence of chlamydospore or pseudohyphae formation^10,34^.

#### Tolerance of NaCl concentration

The ability of *C. auris* to tolerate high salt concentrations was tested. For this purpose, all isolates were cultured on SDA media with 10% NaCl and incubated at 37 °C for 48 h. For comparison, *C. famata* and *C. albicans* were cultured under the same conditions^35^.

#### Hemolytic activity

All *C. auris* isolates were cultured on blood agar plates prepared by adding 7 mL of sheep blood to 100 mL of SDA supplemented with 3% glucose. The final pH of the medium was 5.6 ± 0.2. The plates were incubated at 37 °C in aerobic and anaerobic conditions (5% CO_2_) for 48 h. The results were recorded as a distinct clear zone around the colonies as evidence for the lysis of red blood cells^36^.

#### Phospholipase activity

Phospholipase activity of the *C. auris* strains was assessed using SDA supplemented with 14.6 g NaCl and 0.138 g CaCl_2_. Following autoclave sterilization and cooling to approximately 50 °C, 50 mL of egg yolk emulsion was added. An inoculum of 10 μL containing 1 × 10^7^ yeast cells/mL was spotted at the center of each plate. All the plates were then incubated at 37 °C for five days. Phospholipase production was indicated by the formation of a whitish precipitation zone surrounding the colonies^36^.

#### Protease activity

To assess protease activity, a specific agar medium was prepared containing 0.04 g MgSO_4_·7 H_2_O, 0.5 g K_2_HPO_4_, 1 g NaCl, 0.2 g dried yeast extract, 4 g glucose, and 0.05 g bovine serum albumin mixed with 180 mL SDA. Protease activity was considered positive when forming a precipitation zone surrounding the colonies^36^.

#### Esterase activity

Esterase activity was tested using a medium composed of 10 g peptone, 5 g NaCl, 0.1 g CaCl_2_, and 15 g of agar dissolved in 1000 mL of distilled water. After sterilization, 5 mL of autoclaved Tween 80 was added. A 10 µL of *C. auris* suspension (10^6^ cells/mL) was spot inoculated on plates and incubated at 30 °C. Esterase production was indicated by precipitation halo surrounding the colonies when viewed under transmitted light^36^.

#### Evaluation of Virulence enzymes

For hemolysin, lipase, protease and esterase activity index, the diameter of colony and precipitation zone were measured, and the activity was calculated using the following formula:

Pz: *colony diameter/colony diameter with precipitation zone diameter*

The results were classified as non-activity (Pz = 1), weak (0.64 < Pz < 0.99), and strong activity (Pz ≤ 0.63)^36^.

#### Determination of biofilm formation

*Qualitative assessment of biofilm production* The medium is composed of BHI (37 gm/L), glucose (80 gm/L), and agar (10 gm/L). Congo red was prepared as a concentrated aqueous solution and autoclaved at 121 °C (Congo red stain 0.8 gm/L) for 15 min, separately from other medium constituents. It was then added when the agar had cooled to 55 °C^37^. Plates were inoculated and incubated aerobically for 24–48 h at 37 °C. Dark red colonies indicated positive results.

*Quantitative assessment of biofilm production* Individual wells of sterile 96 well-flat bottom microtiter plates were filled with 100 μl aliquots of the cell suspension. The plates were then incubated at 37 °C for 72 h. After incubation, the plates were tapped to gently remove each well’s content. Plates were stained with 0.1% (w/v) crystal violet, followed by thorough rinsing with deionized water to remove excess stain. The plates were then left to dry. The optical density (OD) of the stained adherent biofilm was measured at 492 nm using an ELISA reader. Based on the mean OD values, isolates were categorized into three groups: strong biofilm producers (OD > 0.320), moderate biofilm producers (OD between 0.120 and 0.320), and non or weak biofilm producers (OD < 0.120)^38^. A biofilm-producing *C. albicans* was used as the positive control, while the negative control consisted of a non-biofilm-producing yeast, *Nakaseomyces glabratus* (formerly *C. glabrata*).

### Statistical analysis

Data were analyzed using the statistical package for social sciences, version 20.0 (SPSS Inc., Chicago, Illinois, USA). Quantitative data were expressed as mean ± standard deviation (SD). Qualitative data were expressed as frequency and percentage. A Chi-square (× 2) test of significance was used to compare the proportions between the qualitative parameters. While Fisher’s exact test was used to evaluate the relation between two groups of categorical variables. Cohen’s Kappa coefficient (κ) was determined as a measure of agreement between different variables while testing interrater reliability by comparing VITEK 2 to CLSI method of antifungal agents. The confidence interval was 95%, and the accepted margin of error was 5%. So, the *P* value was considered significant as the following probability value < 0.05 was considered significant; < 0.001 was considered highly significant; and *P* value > 0.05 was considered insignificant. Statistical comparisons were made by analysis of variance (one-way ANOVA) followed by a Tukey–Kramer post hoc test. Two-way ANOVA was used to indicate the difference between *C. auris* virulence factors and associated clinical manifestations, and Tukey’s test for multiple comparisons was used as a post hoc test.

## Results

The results summarize the findings obtained from the analysis of *Candidozyma auris* (formerly *Candida auris*) recovered during the study period. They describe the distribution of isolates across different specimen types and patient demographics, the reliability of identification methods, antifungal susceptibility profiles, and the expression of different virulence factors. The data offer preliminary insights into the potential characteristics and clinical relevance of *C. auris* in the studied population.

This study recovered 140 antifungal-resistant *Candida* isolates from clinical specimens (Table 1). *Candidozyma auris* was the most frequently identified species, accounting for 120 isolates (85.7%). Other *Candida* species included *C. albicans* (6 isolates; 4.3%), *C. tropicalis* (6 isolates; 4.3%), *C. famata* (4 isolates; 2.85%), and *Nakaseomyces glabratus* (4 isolates; 2.85%).

**Table 1:** Distribution of *Candida* species isolated from clinical specimens (n = 140).

*Candida* species	Number of isolates (n)	Percentage (%)
*Candida auris*	120	85.7
*Candida albicans*	6	4.3
*Candida tropicalis*	6	4.3
*Candida famata*	4	2.85
*Nakaseomyces glabratus*	4	2.85

**Fig. 2:**
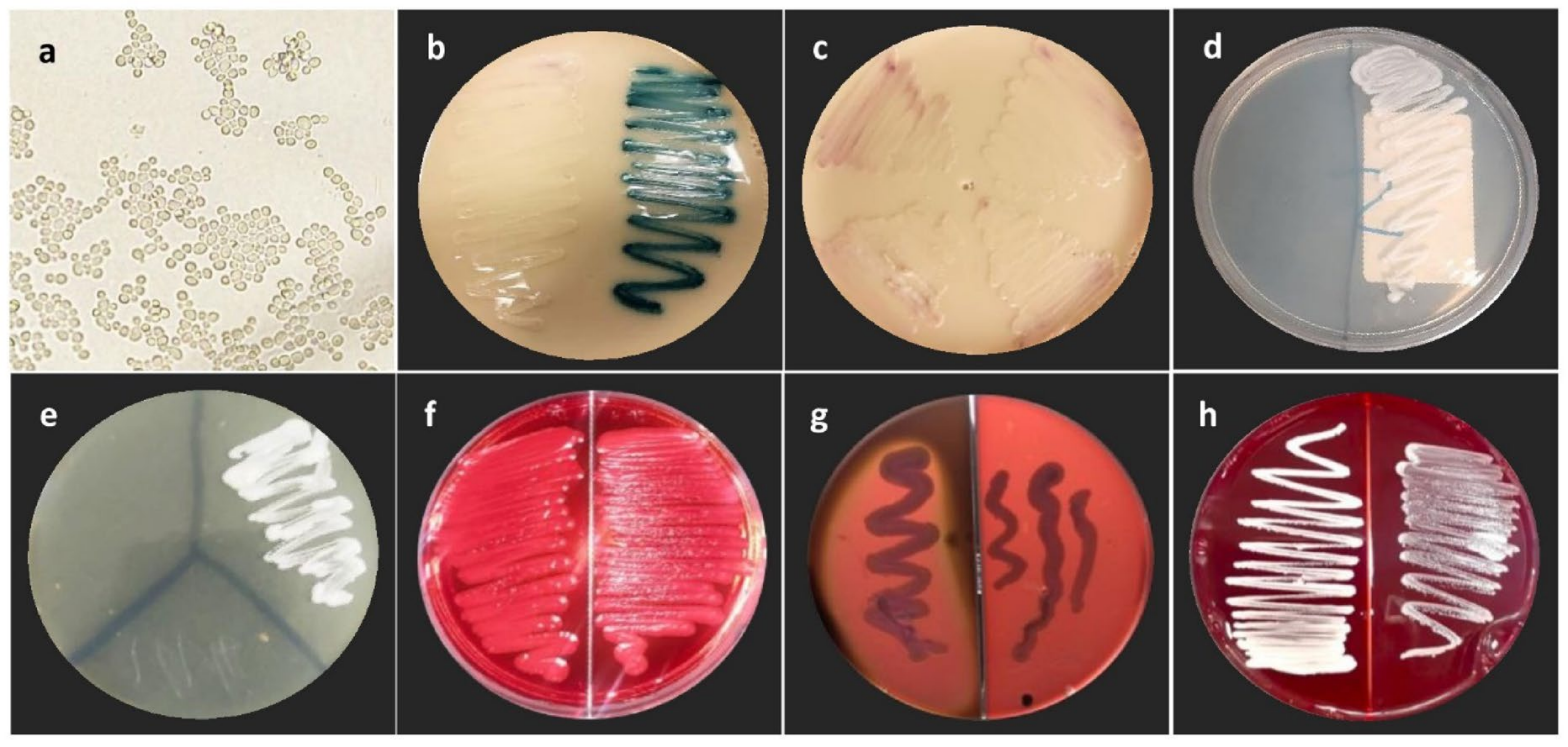
Virulence Factors of *C. auris* (**a**) Micromorphology on chromogenic agar displaying budding yeast cells of *C. auris*. (**b**, **c**) Color variation of *C. auris* colonies on chromogenic agar, ranging from white to light pink, in contrast to the characteristic green coloration of *C. albicans*. (**d**) Growth at 42 °C: *C. auris* (right) shows growth, whereas *C. albicans* (left) does not. (**e**) Growth in 10% NaCl medium: only *C. auris* demonstrates growth, while *C. famata* and *C. albicans* show no growth. (**f**) Positive biofilm formation by *C. auris* indicated by red colonies on Congo red agar. (**g**) Beta-hemolysis by *C. auris* under anaerobic conditions. (**h**) Absence of hemolysis by *C. auris* under aerobic conditions.

The recovered *Candida* isolates were identified using Vitek 2® Compact, MALDI-TOF, and chromogenic media. The results indicated that both MALDI-TOF and chromogenic agar yielded identical identification outcomes, In contrast, VITEK 2 demonstrated inconsistencies, as it failed to identify some isolates correctly, suggesting that 20 isolates were misidentified as *C. auris* (Table 2). *C. auris* strains demonstrated robust growth at 42 °C, confirming their thermotolerance. On chromogenic agar, *C. auris* typically produced white to pale pink colonies (Fig. 2). Green colonies were indicative of *C. albicans*, dark blue colonies suggested the presence of *C. tropicalis*, and beige colonies were consistent with *N. glabratus*. *C. famata* forms off-white to beige colonies with colony edges that may appear purplish or deep pink. This color tones may seem similar to *C. auris* and potentially misleading, but an experienced eye can readily distinguish between their growth patterns.

**Table 2:** Comparison between different identification methods in the detection of *C. auris.*

	Candida species identified				
	*C. auris*	*C. albicans*	*C. tropicalis*	*C. famata*	*N. glabratus*
Vitek 2	140	0	0	0	0
MALDI-TOF	120	6	6	4	4
CHROM gar	120	6	6	4	4

According to the EORTC/MSG criteria^22^, all cases with *C. auris* showed consistent clinical and radiological evidence of infection and were categorized as proven infection. When analyzing the demographic data, it was found that among the 120 patients with *C. auris* isolates, 72 (60%) were males and 48 (40%) were females. The majority of cases (71; 59%) occurred in patients aged 60 years or older, while 49 (41%) were under 60 years of age (Table 3). In the present study, *C. auris* cases were classified into two main groups; candidemia (60.8%), which represents the majority of infections, and non-candidemia cases, which included urinary tract infections (15%), respiratory tract infections identified from sputum samples (12.5%) and bronchoalveolar lavage (BAL) samples (8.3%), as well as isolates from drain fluids (2.5%) and tissue samples (0.8%).

**Table 3:** Demographic characteristics, clinical specimen sources, and associated risk factors of patients with *C. auris* isolates (n = 120).

Category				No. (%)
Gender	Male			72 (60.0%)
	Female			48 (40.0%)
Age group	< 60 years			49 (41.0%)
	≥ 60 years			71 (59.0%)
Specimen source	Candidemia		Blood	73 (60.8%)
	Non-candidemia	Urinary tract	Urine	18 (15.0%)
		Respiratory tract	Sputum	15 (12.5%)
			BAL	10 (8.3%)
		Intraabdominal	Drain	3 (2.5%)
			liver biopsy	1 (0.8%)
Risk factors	Transplant			43 (35.8%)
	Post COVID-19			37 (30.8%)
	Cancer			30 (25.0%)
	Diabetes mellitus (DM)			5 (4.2%)
	Renal failure (RF)			4 (3.3%)
	Pneumonia			1 (0.8%)

Analysis of associated risk factors revealed that transplantation was the most frequent underlying condition (35.8%), followed by post-COVID-19 history (30.8%), cancer (25%), diabetes mellitus (4.2%), renal failure (3.3%), and pneumonia (0.8%).

Regarding the clinical outcomes of the 120 cases, death was confirmed in 47 patients, while the status of the remaining patients could not be recorded due to inability to follow-up. Candidemia cases showed the highest mortality rate (39/47 deaths, 82.9%), followed by patients with isolates from sputum (4/47, 8.5%), urine (2/47, 4.3%), and bronchoalveolar lavage (BAL) (2/47, 4.3%). Clinical outcome data were not available for surgical drains and tissue biopsy patients.

The in vitro activity of nine antifungal agents against *C. auris* isolates is summarized in Table 4. Minimum inhibitory concentrations (MICs) were presented as MIC_50_, MIC_90_, range, median, and geometric mean values, all in µg/mL. Interpretation was guided by tentative breakpoints proposed by the CDC^28^, previously published epidemiological cutoff values (dECOFFs)^29,30^ and previously published studies^31–33^. The number and percentage of isolates exceeding these thresholds were reported to provide context for potential reduced susceptibility.

**Table 4:** Antifungal susceptibility of *C auris* isolates (n = 120).

	MIC_50_ (µg/mL)	MIC_90_ (µg/mL)	Range (µg/mL)	Median	GM (µg/mL)	Resistant	Susceptible n (%)	*P* value
FLC ≥ 32 µg/mL^28^	32	32	8–64	32	26.63	120 (100%)	0 (0%)	< 0.001
VOR 1 µg/mL^29,30^	0.125	0.5	0.25–1	0.5	0.425	0 (0%)	120 (100%)	< 0.001
POSA 0.125 µg/mL^29,30^	0.03	0.125	0.015–0.125	0.03	0.034	0 (0%)	120 (100%)	< 0.001
ITRA 0.25 µg/mL^29,30^	1	4	0.5–4	1	1.282	120 (100%)	0 (0%)	< 0.001
KETO ≥ 1 µg/mL^32,33^	16	64	4–64	16	16.95	120 (100%)	0 (0%)	< 0.001
AMB ≥ 2 µg/mL^28^	0.5	1.0	0.125–1	0.5	0.51	0 (0%)	120 (100%)	< 0.001
CAS ≥ 2 µg/mL^28^	0.25	1.0	0.25–2	0.25	0.31	4 (3.3%)	116 (96.7%)	< 0.001
MICF ≥ 4 µg/mL^28^	0.125	0.5	0.125–4	0.12	0.199	5 (4.2%)	115 (95.8%)	< 0.001
FCT ≥ 32 µg/mL^25,31^	64	128	8–128	64	58.01	116 (96.7%)	4 (3.3%)	< 0.001

GM, geometrical mean; FLC, fluconazole; VOR, voriconazole; POS, posaconazole; ITRA, itraconazole; KETO, ketoconazole; AMB, amphotericin b; CASP, caspofungin; MFG, micafungin; FCT, 5-flucytosine, Highly significant (*P* ≤ 0.001).

All *C. auris* isolates displayed fluconazole MIC_50_/_90_ values of 32/32 µg/mL, with the entire population falling at or above the CDC’s proposed breakpoints (≥ 32 µg/mL). Voriconazole and posaconazole MICs remained within their respective wild-type distributions as defined by published dECOFFs. Itraconazole MICs for all isolates exceeded the proposed dECOFF of 0.25 µg/mL for *C. auris* for this drug, with values ranging from 0.5 to 4 µg/mL. Amphotericin B MICs ranged from 0.125 to 1.0 µg/mL, with no isolates exceeding the CDC’s tentative breakpoint of ≥ 2 µg/mL. Caspofungin and micafungin showed elevated MICs in a small subset of isolates (3.3% and 4.2%, respectively), with MICs reaching the tentative cutoff values ≥ 2 and ≥ 4 µg/mL, respectively.

Flucytosine MICs ranged from 8 to 128 µg/mL, with 96.7% of the isolates exceeding CLSI breakpoint of ≥ 32 µg/mL used for other *Candida* species, suggesting limited activity. Ketoconazole, for which no standardized breakpoints exist, showed high MICs across all isolates (MIC_50_/_90_ = 16/64 µg/mL), and all values were ≥ 1 µg/mL, consistent with previous reports of reduced in vitro activity in other *Candida* species. *P* values represent comparisons of categorical susceptibility outcomes (resistant vs. susceptible) for each antifungal agent.

The agreement between CLSI BMD and VITEK 2 for antifungal susceptibility testing was evaluated across all the isolates for fluconazole, voriconazole, amphotericin B, caspofungin, and micafungin (Table 5). For fluconazole, all isolates had MICs above the CDC breakpoint, with 100% agreement between methods. For voriconazole, 14 isolates (12%) were classified above the resistance breakpoint. The EA and CA between VITEK 2 and BMD were 71% and 88%, respectively, with a Cohen’s kappa of 0.88, reflecting strong agreement. For amphotericin B, 23 isolates (19%) had MICs above the proposed epidemiological cutoff values (ECVs). The EA was 65%, while CA reached 80%. The corresponding Cohen’s kappa was 0.80, indicating substantial agreement. In the case of caspofungin and micafungin, 4 isolates (3%) showed elevated with a Cohen’s kappa of 0.96, denoting almost perfect agreement. Flucytosine showed a relatively low EA at 54% between testing methods and a lower CA at 35%, indicating substantial inconsistency in susceptibility categorization. Furthermore, Cohen’s kappa value of 0.35 reflects only fair agreement, suggesting that the reliability of susceptibility testing for flucytosine in this study is limited and should be interpreted with caution. Due to variability observed in the VITEK 2 results, the CLSI BMD method was adopted to classify the isolates according to their antifungal susceptibility.

**Table 5:** Agreement between VITEK 2 and CLSI broth microdilution for antifungal susceptibility testing of *C. auris* isolates.

Antifungal agent	FLC	VOR	AMB	CAS	MICF	FCT
Isolates (n)	120	120	120	120	120	120
Resistant (n)	120 (100%)	14 (12%)	23 (19%)	4 (3%)	4 (3%)	78 (65%)
Susceptible (n)	0	106	97	116	116	42
EA (%)	78	71	65	99	97	54
CA (%)	100	88	80	96	96	35
Cohen’s kappa	1	0.88	0.8	0.96	0.96	0.35

FLC, fluconazole; VOR, voriconazole; AMB, amphotericin b; CASP, caspofungin; MFG, micafungin; FCT, 5-flucytosine; CA, categorical agreement; EA, essential agreement, ≤ 0 as no agreement, 0.01–0.20 as slight, 0.21–0.40 as fair, 0.41–0.60 as moderate, 0.61–0.80 as substantial, and 0.81–1.00 as almost perfect.

Microscopic examination on cornmeal agar revealed that the isolates consistently exhibited unicellular budding yeast morphology. No pseudohyphae, hyphae, or chlamydospores were observed under the test conditions (Fig. 2). *C. auris* isolates showed tolerance to high salt concentrations. Growth was robust on SDA media supplemented with 10% NaCl, comparable to that of *C. albicans* and *C. famata* under the same conditions (Fig. 2). This underscores the high osmotic tolerance of the recovered *C. auris* isolates.

Lytic enzymes involved in *C. auris* pathogenicity were assessed. On glucose-enriched blood agar, *C. auris* isolates exhibited clear beta hemolysis under anaerobic conditions, this indicates the active secretion of hemolysins capable of complete hemolysis in the absence of oxygen. No hemolytic activity was observed under aerobic conditions (Fig. 2).

The evaluation of virulence enzymatic activity among *C. auris* isolates revealed varying strengths of production across the four enzymes (Table 6). Protease (Pz) showed the highest proportion of strong producers, with 96 isolates (80%) demonstrating strong activity, and a statistically significant difference in production strength (*P* = 0.0007). Hemolysin (Hz) also displayed a strong production in 89 isolates (74.2%) (*P* = 0.0013). Likewise, esterase (Ez) had 82 strong producers (68.3%) and 5 non-producers (4.2%), showing significance in activity strength (*P* = 0.0340). Although lipase (Lz) had the highest number of strong producers (101 isolates, 84.2%), the difference in production strength was not statistically significant (*P* = 0.1447), suggesting a more consistent expression across isolates.

**Table 6:** The strength of enzymatic activity among *C. auris* clinical isolates.

	Mean (mm) ± SD	Strong n (%)	Weak n (%)	Non-producer n (%)	*P* value
Protease (Pz)	0.54 ± 0.17	96 (80%)	21(17.5%)	3 (2.5%)	0.0007**
Hemolysin (Hz)	0.49 ± 0.23	89 (74.2%)	31(25.8)	0 (0%)	0.0013*
Esterase (Ez)	0.59 ± 0.21	82 (68.3%)	33 (27.5%)	5 (4.2%)	0.0340*
Lipase (Lz)	0.53 ± 0.16	101 (84.2%)	16 (13.3%)	3 (2.5%)	0.1447^ns^

**P < 0.001 = Highly significant; *P < 0.05 = significant; ^ns^*P* > 0.05 = Non-significant.

**Fig. 3:**
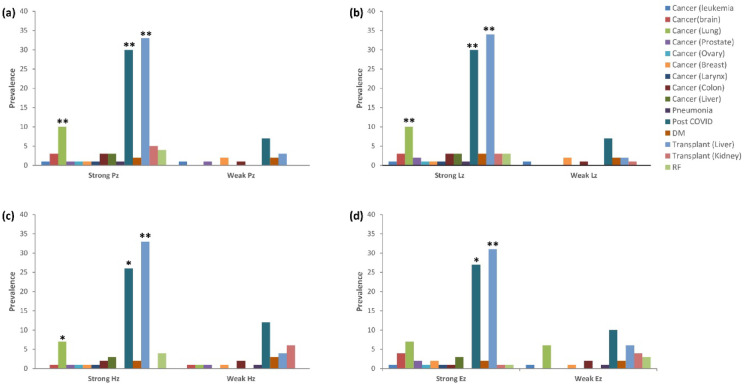
Association between *C.auris* virulence enzyme activity and patients’ underlying conditions. (**a**) Protease activity (Pz), (**b**) Lipase activity (Lz), (**c**) Hemolysin activity (Hz), and (**d**) Esterase activity (Ez), **highly significant, *Significant (p < 0.05).

The relationship between enzymatic activity and underlying clinical conditions was investigated across *C. auris* isolates (Fig. 3). Lipase (Lz) and protease (Pz) activities showed significantly high associations with liver transplantation, post-COVID-19, and lung cancer. Similarly, hemolysin (Hz) activity demonstrated a strongly significant association with liver transplantation, while significant associations were also observed in isolates from post-COVID-19 and lung cancer patients. In contrast, esterase (Ez) activity demonstrated a high level of statistical significance with liver transplantation and a significant association with post-COVID-19. No significant associations were observed between weak enzyme production and any of the underlying conditions across all enzymes.

**Fig. 4:**
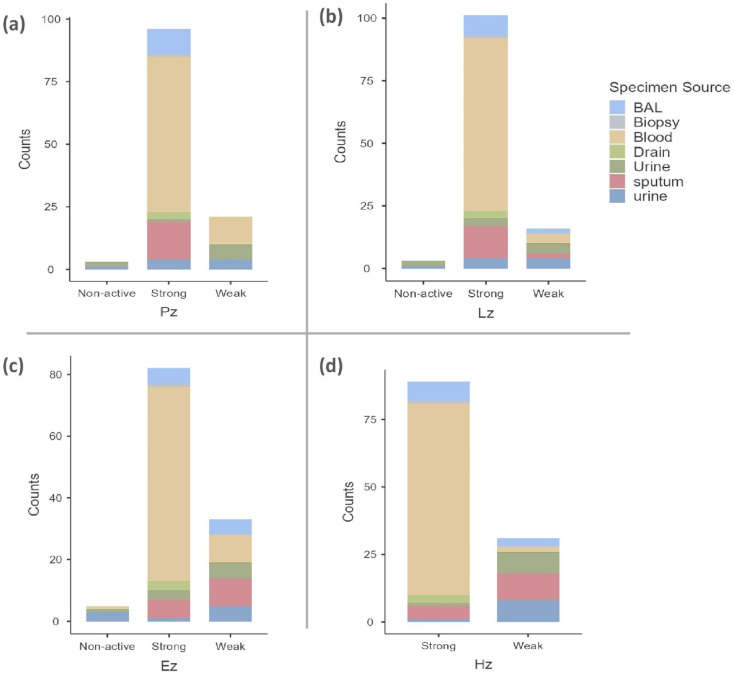
Distribution of enzyme activity of *C. auris* across different clinical specimen types. (**a**) Protease (Pz), (**b**) Lipase (Lz), (**c**) Hemolysin (Hz), and (**d**) Esterase (Ez).

Activity levels and specimen source for all enzymes were examined (*P* < 0.001 for each) (Fig. 4). Across all enzymes, strong activity was most frequently associated with blood specimens, suggesting a consistent pattern of higher enzyme expression in bloodstream-derived isolates. In contrast, weak activity was more commonly observed in urine and sputum samples, particularly for protease, esterase, and hemolysin. Non-active isolates were infrequent overall, but when present, they were more likely to originate from non-blood sources, especially urine. The differences in distribution between observed and expected frequencies indicate that the specimen source plays a role in the expression pattern of these enzymes. While the association strength varied among enzymes, the relationship was particularly marked for hemolysin and esterase (Cramer’s V: 0.453 for protease, 0.453 for lipase, 0.475 for esterase, 0.776 for hemolysin), showing deviations across sample types.

**Fig. 5:**
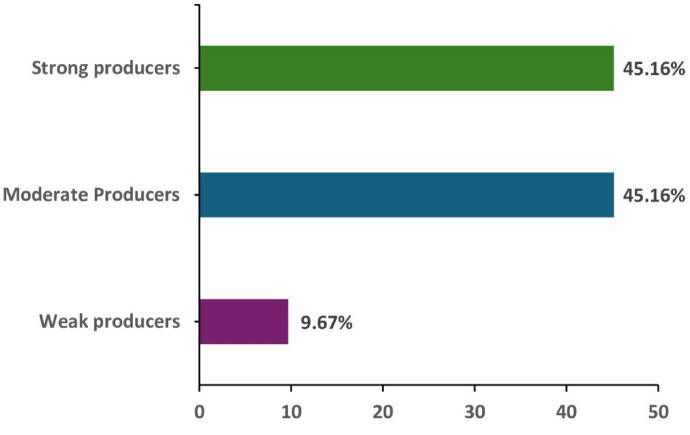
Biofilm production among *C. auris* strains.

Assessment of biofilm formation among 31 *C. auris* isolates revealed that the majority exhibited moderate to strong biofilm-producing ability (Fig. 5). Specifically, 45.16% of the isolates were classified as moderate biofilm producers, while an equal proportion (45.16%) demonstrated strong biofilm formation. A smaller subset, accounting for 9.67%, were identified as weak biofilm producers.

**Fig. 6:**
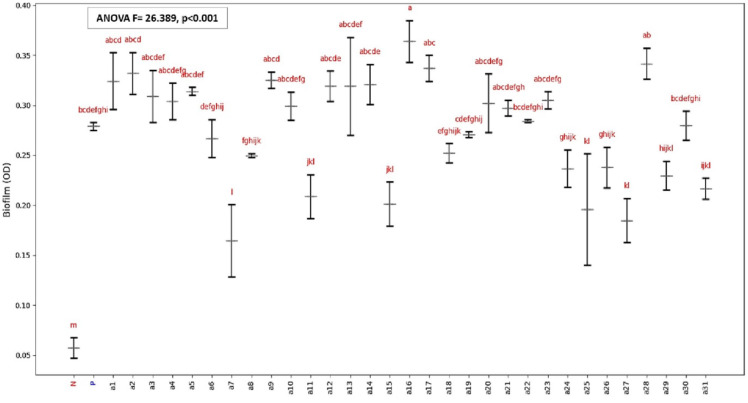
Biofilm production by different *C. auris* strains (a1-a31) represented as mean optical density (OD) ± standard deviation (SD). “P” and “N” represent the positive (*C. albicans*) and negative (*N. glabratus*) controls, respectively. Red lowercase letters indicate statistical groupings based on post-hoc multiple comparison analysis, groups sharing at least one letter are not significantly different (p < 0.05).

The biofilm production of *C. auris* strains (a1–a31) compared to *C. albicans* (positive control) and *N. glabratus* (negative control) was evaluated (Fig. 6). Statistical analysis confirmed significant difference between the groups (*P* < 0.05). The results, expressed as mean optical density (OD) readings ± standard deviation (n = 3), revealed significant variability in biofilm production among the strains. The strongest biofilm producer among *C. auris* strains was a16 (0.3640 ± 0.02095), outperforming the positive control (0.2790 ± 0.00400). In contrast, the weakest *C. auris* biofilm producers were a27 (0.1847 ± 0.02203) and a25 (0.1957 ± 0.05572), though these were still significantly stronger than the negative control (0.0573 ± 0.01026).

## Discussion

This study provides a preliminary overview of *Candidozyma auris* (formerly *Candida auris*) in a tertiary care setting in Egypt. It highlights key aspects such as epidemiology, identification challenges, antifungal resistance, and virulence. Subjects were patients with proven *C. auris* infections that exhibited resistance to at least one class of antifungal agents. In this study, the distribution of antifungal-resistant *Candida* species revealed a predominance of *C. auris*, accounting for 85.7% of the total 140 isolates. This finding supports the growing global concerns regarding the emergence and dominance of *C. auris* as a significant multidrug-resistant pathogen. Recent studies have reported *C. auris* as an increasingly common cause of healthcare-associated invasive candidiasis, often surpassing traditional *Candida* species in certain regions^39,40^.

Regionally, *C. auris* is increasingly reported across the Middle East and North Africa, though surveillance and diagnostic gaps remain significant. Initial cases in the region were documented in the UAE, Kuwait, and Saudi Arabia, and often linked to outbreaks and bloodstream infections^41^. Across Africa, *C. auris* has been confirmed in six countries: South Africa, Kenya, Nigeria, Sudan, Algeria, and Egypt. The predominant genetic lineages identified include Clade III (African) followed by Clade I (South Asian), with other clades also reported, suggesting multiple introductions^42^. The first confirmed case in Egypt was reported in 2017 in a patient who had returned from Saudi Arabia, suggesting likely colonization abroad. The isolate was identified and then classified as Clade I (South Asian)^43^. Despite initial concern, subsequent surveillance did not detect further cases at that time^43^. In 2024, *C. auris* was identified in 3.6% of clinical *Candida* isolates, suggesting its local circulation in Egyptian healthcare settings^45^. More recent isolates from Alexandria further support its ongoing presence in Egypt^46^.

The predominance of *C. auris* observed in this study may be attributed to its unique ability to persist in hospital environments and its rapid nosocomial transmission. This suggests that *C. auris* is displacing other antifungal-resistant *Candida* in certain settings. This indicates a need for revised treatment approaches and infection control measures. Management strategies can differ significantly between *Candida* species, given the distinct resistance profile of *C. auris*.

Accurate identification is essential for controlling the dissemination of *C. auris*, as it enables targeted interventions and control of this multidrug-resistant pathogen^47^. However, limitations in commonly used diagnostic systems pose significant challenges. The lack of detection of *C. auris* in the Middle East and North Africa is more likely due to technical limitations rather than the true absence of the pathogen^41,42^. Many regional tertiary hospitals continue to rely on conventional methods, which increases the likelihood of misidentification and underreporting of *C. auris*^41,45^.

In the current study, multiple identification methods were employed in accordance with the CDC’s *C. auris* identification algorithms^47^. The VITEK 2 yeast identification system showed lower accuracy in identifying *C. auris* even when using updated databases^24,48^. This system primarily relies on phenotypic identification, a limitation that becomes more evident with *Candida* species due to their phenotypic similarities and the genetic diversity within different clades. A study evaluating VITEK 2 system (software version 8.01) found that the system correctly identified *C. auris* in only 52% of cases^48^. Misidentification of *C. auris* as closely related species, such as *Candida haemulonii* and *Candida duobushaemulonii*, has been previously reported^24,48^. In the current findings, misidentification occurred between *Candida famata*, *C. albicans*, *C. tropicalis*, and *Nakaseomyces glabratus.* This would affect appropriate treatment and control due to differences in virulence, antifungal resistance profiles, and clinical management strategies.

In contrast, MALDI-TOF demonstrated superior accuracy, reliably distinguishing *C. auris* from other species, especially when using updated databases. The accuracy of this method is due to its ability to analyze unique protein profiles, making it a gold standard for *C. auris* identification in clinical microbiology laboratories^26,49^. In contrast, chromogenic media lacks molecular specificity and may not be completely reliable for the accurate identification of *C. auris*. However, they offer a simple and low-cost preliminary tool in the absence of *C. auris*-specific identification kits, especially when combined with growth at 42 °C^23,25^. This approach is particularly valuable in resource-limited healthcare settings, where access to advanced methods such as MALDI-TOF or molecular diagnostics is often restricted or cost-prohibitive^25^.

The demographic analysis of *C. auris* cases revealed that the infection was more prevalent among males and patients aged 60 or older, which aligns with previous reports^11,50,51^. This demographic pattern may reflect age-related immunosuppression, comorbidities, and differences in healthcare exposure among genders. The classification of *C. auris* cases into candidemia (60.8%) and non-candidemia infections further supports the pathogen’s role as a primary bloodstream infectious agent outstanding other species such as *N. glabratus*^53^. Recorded non-candidemia infections such as urinary tract, respiratory tract, and intraabdominal infections demonstrate the ability of *C. auris* to colonize various anatomical sites. This capability has been well documented in nosocomial outbreaks^50,51^.

Analysis of risk factors identified transplantation as the most common underlying condition, followed by a history of post-COVID-19 infection, cancer, diabetes mellitus, renal failure, and pneumonia. These findings align with previous studies showing that *C. auris* affects immunocompromised patients, particularly those receiving immunosuppressives or experiencing prolonged hospital stays^50,51^. Procedures as catheterization can also create potential entry points for fungal colonization and bloodstream invasion^53,54^. Moreover, COVID-19 pandemic has significantly contributed to the increased prevalence of *C. auris* infections due to mechanical ventilation, and the widespread use of corticosteroids and broad-spectrum antibiotics^13,52^.

Our results concerning the antifungal susceptibility profiles for *C. auris* provide important insights into the resistance patterns observed in Egypt. The resistance to fluconazole (100%) and the reduced activity of flucytosine (96.7%) is consistent with global data showing that *C. auris* is intrinsically resistant to fluconazole due to mutations in the *ERG11* gene^55^. Regional studies across the Middle East and Africa also demonstrated universal resistance to fluconazole^41,42^, that was similarly observed in the first Egyptian isolate reported by Khairat et al.^44^.

The low minimum inhibitory concentration (MIC) values of amphotericin B observed in this study is noteworthy, as global reports indicated varying resistance levels to this drug. While approximately 30% of *C. auris* isolates in the United States have shown resistance to amphotericin B^26^, a recent study from Egypt by Mahmoud et al. (2024) reported universal resistance among bloodstream isolates. This discrepancy may reflect clade-related differences, strain-specific variability, or local antimicrobial use patterns^28^. Consistent with the literature from Africa^42^, elevated MICs to itraconazole, ketoconazole, and flucytosine were also noted. In contrast, the reduced MICs for caspofungin (96.7%) and micafungin (95.8%) in our isolates support their continued role as first-line agents. However, the detection of resistant isolates (3.3% and 4.2%, respectively) aligns with the emerging global and regional reports of reduced echinocandin susceptibility due to mutations in the *FKS1* gene^41,42,55,56^. A previous study reported that the key mutations, such as Y132F in *ERG11* (conferring azole resistance) and F635Y in *FKS2* (linked to echinocandin resistance), were consistently identified across some tested strains from Egypt^57^.

Moreover, the agreement between VITEK 2 and CLSI BMD across multiple antifungal classes was evaluated. The VITEK 2 system demonstrated high levels of categorical agreement (CA) and essential agreement (EA) with the CLSI BMD method for most antifungal agents. Agreement was excellent for fluconazole, caspofungin, and micafungin. Voriconazole and amphotericin B showed slightly lower agreement levels. In contrast, Siopi et al. reported poor agreement for fluconazole (69%) and amphotericin B (31%) using 100 international *C. auris* isolates from different clades. These differences may be attributed to geographic variation in isolate susceptibility profiles^58^. Comparing both data sets on 5-flucytosine, limited reliability in 5-flucytosine susceptibility testing using automated methods was observed. Interestingly, both studies support the accuracy of VITEK 2 in detecting echinocandin susceptibility. However, careful interpretation is needed for azole, 5-flucytosine, and amphotericin B results.

In addition to its concerning resistance patterns, the virulence attributes of *C. auris* are still not fully understood. While phenotypic switching and morphogenesis are well-known virulence factors of some *Candida* species^59^, *C. auris* rarely produce germ tubes, pseudohyphae, or chlamydospores^9^. This was confirmed in an infection model in *Galleria mellonella*, during which the isolates did not undergo significant filamentation at any time post-infection^60^. However, Recent studies have identified phenotypic switching system in *C. auris* induced by passage through a mammalian host, culturing on CHROMagar *Candida,* genotoxic stressors and high-salt stress^61–63^. In our study, none of *C. auris* isolates formed chlamydospores or pseudohyphae after growth on cornmeal agar and incubation for 5 days at 30 °C. Usually, *C. auris* lacks candidalysin and hyphal cell wall protein, which are essential for full hyphal growth^11^. However, the lack of pseudohyphae does not necessarily indicate reduced virulence, as *C. auris* exhibits other virulence factors such as biofilm formation and enzyme production. Overall, the absence of pseudohyphae on cornmeal agar is a notable feature of *C. auris* but should be considered along with other diagnostic criteria for reliable identification^62^.

*Candidozyma auris* exhibits thermotolerance, growing optimally at 37 °C and maintaining viability up to 42 °C^62^. The ability of *C. auris* strains to grow at 42 °C highlights the thermotolerance of this emerging pathogen. In addition to being a useful diagnostic feature^23^, thermotolerance is an important virulence factor contributing to the ability of *C. auris* to survive various temperature gradients in the host’s body, causing invasive infections^64^. Notably, proteases activity produced by *C. auris* was found significantly higher at 42 °C, which means that the pathogenicity of *C. auris* is conserved even at such high temperatures^61^. This may explain its persistence in hospital environments, potentially complicating infection control measures^64^. Moreover, some researchers propose that global warming is linked to *C. auris* thermotolerance. It is suggested that *C. auris* may be the first example of a new pathogenic fungus emerging from global warming^65^. Furthermore, all *C. auris* strains were also found to be halotolerant. The halotolerance of *C. auris* enables it to survive on the skin, particularly in the axilla and groin, which are exposed to frequent sweating and high temperatures^13^.

Multiple studies have highlighted the virulence potential of *C. auris* through the activity of proteases, hemolysins, lipases, and esterases. Proteases has been linked to tissue invasion and immune evasion^11,66,67^. Lipases also contribute to biofilm formation, host cell damage, and immune evasion. Besides, hemolysin plays a key role in *C. auris* virulence by facilitating iron sequestration, which supports rapid growth and dissemination^11^. Although its characterization in *Candida* species remains inconsistent, esterases assist in host cell adhesion and suppression of competing microflora^68^.

The assessment of virulence-associated enzymatic activity in *C. auris* isolates demonstrated distinct patterns in expression strength across the four investigated enzymes. Protease, hemolysin, and esterase exhibited statistically significant variability in activity, with a predominance of strong producers. In contrast, lipase did not show any significance. Production of virulence enzymes appears to be clade or strain dependent. For example, some studies reported limited activity of lipases; only detected in 37.5% of the strains with weak activity^10,66^. In contrast, Colombian clinical isolates showed high levels of protease and hemolysin activity, with 68% producing hemolysin and nearly all exhibiting protease activity^66^. Another recent study identified weak exoenzyme activity among all the isolates, with minimal hemolytic or proteinase activity, implying significant geographical and clade variability^36^.

Our data suggested that the strong enzymatic activity, particularly of hemolysin, lipase, and protease, was more frequently associated with some high-risk conditions such as liver transplantation, COVID-19, and lung cancer. Conversely, weak enzyme expression did not appear to correlate significantly with any underlying condition. The presence of robust enzyme activity has been correlated with more severe outcomes in invasive infection models, especially in immunocompromised host^66^. However, direct clinical correlation with patient comorbidities remains limited in published literature.

The enzymatic activity of *C. auris* varied significantly with specimen source, with strong activity linked to blood isolates and weaker or absent activity more common in urine and sputum. This pattern underlines a potential relationship between enzyme expression and infection sites. A recent study suggested possible associations between virulence and clinical origin of *C. auris*. Blood isolates were more virulent than those isolated from urine and respiratory specimens in *G. mellonella* model of candidiasis^60^.

Hemolysin production in *Candida* species is influenced by some factors as oxygen availability, nutrient levels, and species-specific traits^69–71^. While *C. albicans* shows strong oxygen dependency with only 5% of isolates hemolyzing under anaerobic conditions, non-*albicans* species retain significant hemolytic activity in both aerobic (88%) and anaerobic (68%) environments^71^. These differences suggest that oxygen levels may affect virulence in low-oxygen tissue sites. Additionally, glucose-enriched environments have been shown to enhance hemolysin production in several *Candida* species, linking increased metabolic activity to greater virulence potential, particularly under hyperglycemic conditions^70,72^.

The present study evaluated the biofilm-forming abilities of *C. auris*. *Candida albicans* tends to form more frequent and stronger biofilms than *C. auris*, likely because they possess a greater number of adhesin-related genes which are comparatively less abundant in *C. auris*^11^. The findings demonstrated the considerable biofilm-forming capacity of *C. auris* strains, with some exceeding *C. albicans*. The capacity of *C. auris* to form biofilms varies significantly among different strains and is influenced by genetic, phenotypic, and regulatory factors^9,73–75^. Further, genetic and molecular analyses have shown that differences in the copy number of adhesin genes contribute to isolate-specific differences in adherence and biofilm formation^74^.

Overall, the findings underscore significant strain-specific variability in the virulence traits of *C. auris*. However, the study’s single-center design and lack of comprehensive genetic characterization represent key limitations. A multicenter investigation with a larger sample size may have uncovered further deviations from the patterns commonly observed in other regions. It is worth noting that our earlier study investigated antifungal resistance-associated genes in a relatively small set of isolates^57^. However, large-scale molecular typing studies and targeted screening of resistance-associated genes are needed to better elucidate the genetic mechanisms underlying resistance and virulence. Moreover, whole-genome sequencing is essential for accurate clade assignment, which is critical for monitoring the geographic distribution and evolutionary dynamics of *C. auris*.

## Conclusion

This study provides the first detailed report of *C. auris* in Egypt, integrating epidemiology, resistance data, and virulence mechanisms and addressing a critical gap in regional data. Ain Shams University Specialized Hospital serves as referral centers for diverse patient populations, thereby enhancing the epidemiological significance of the study and offering valuable insight into the presence and clinical relevance of this emerging pathogen in the region. The findings provide essential data that can inform local infection control policies, guide clinical decision-making, and support the development of targeted prevention and management strategies in healthcare settings. The study provides a foundational reference for future investigations involving broader surveillance and molecular analysis across multiple institutions. By contributing new regional data, this work adds to the global understanding of *C. auris* epidemiology and emphasizes the need for coordinated international efforts in monitoring and managing this public health threat.

## Data Availability

All data generated or analyzed during this study are included in this published article.

## Abbreviations

CDC: The US Centers for Disease Control and Prevention
WHO: The World Health Organization
Saps: Secreted aspartyl proteases
SDA: Sabouraud’s dextrose agar
PDA: Potato dextrose agar
BHI: Brain heart infusion agar
MALDI-TOF MS: Matrix-assisted laser desorption/ionization time-of-flight mass spectrometry
BMD: Broth Microdilution
CLSI: Clinical Laboratory Standards Institute
EUCAST: European Committee on Antimicrobial Susceptibility Testing
OD: Optical Density
BAL: Bronchoalveolar lavage
DM: Diabetes mellitus
RF: Renal Failure
VOR: Voriconazole
POS: Posaconazole
AMB: Amphotericin B
MFG: Micafungin
FLC: Fluconazole
FCT: Flucytosine
CASP: Caspofungin

## References

[1] Cottrel, C. et al. Clustered cases of *Candida auris* colonization: Roles of the infection prevention and control department and the mycology laboratory in controlling transmission. *Med Mycol.* **63**(4), myaf033. 10.1093/mmy/myaf033 (2025). 40205443 10.1093/mmy/myaf033

[2] Satoh, K. et al. *Candida auris* sp. nov., a novel ascomycetous yeast isolated from the external ear canal of an inpatient in a Japanese hospital. *Microbiol. Immunol.* **53**(1), 41–44. 10.1111/j.1348-0421.2008.00083.x (2009). 19161556 10.1111/j.1348-0421.2008.00083.x

[3] Chowdhary, A. et al. New clonal strain of *Candida auris*, Delhi, India. *Emerg. Infect. dis.* **19**(10), 1670. 10.3201/eid1910.130393 (2013). 24048006 10.3201/eid1910.130393 PMC3810747

[4] Pfaller, M. A., Diekema, D. J., Turnidge, J. D., Castanheira, M. & Jones, R. N. Twenty years of the SENTRY antifungal surveillance program: Results for Candida species from 1997–2016. *Open Forum Infect. Dis.* **6**(1), S79–S94. 10.1093/ofid/ofy358 (2019). 30895218 10.1093/ofid/ofy358 PMC6419901

[5] Cristina, M. L. et al. An overview on *Candida auris* in healthcare settings. *J Fungi.* **9**(9), 913. 10.3390/jof9090913 (2023). 10.3390/jof9090913 PMC10532978 37755021

[6] Centers for Disease Control and Prevention (CDC). *Antibiotic Resistance Threats in the United States, 2019* (U.S. Department of Health and Human Services, CDC, 2019). 10.15620/cdc:82532

[7] World Health Organization. *WHO Fungal Priority Pathogens List to Guide Research, Development and Public Health Action* (World Health Organization, 2022). https://www.who.int/publications/i/item/9789240060241

[8] OYong, K. et al. Attributable mortality and cause of death of *Candida auris* cases, Los Angeles County. *Open Forum Infect Dis*. **10**(2), ofad500–2067. 10.1093/ofid/ofad500.2067 (2023)

[9] Bhargava, A., Klamer, K., Sharma, M., Ortiz, D. & Saravolatz, L. *Candida auris*: A continuing threat. *Microorganisms* **13**(3), 652. 10.3390/microorganisms13030652 (2025). 40142543 10.3390/microorganisms13030652 PMC11946832

[10] Larkin, E. et al. The emerging pathogen *Candida auris*: Growth phenotype, virulence factors, activity of antifungals, and effect of SCY-078, a novel glucan synthesis inhibitor, on growth morphology and biofilm formation. *Antimicrob. Agents Chemother.* **61**(5), 10–128. 10.1128/AAC.02396-16 (2017). 10.1128/AAC.02396-16 PMC5404565 28223375

[11] Watkins, R. R., Gowen, R., Lionakis, M. S. & Ghannoum, M. Update on the pathogenesis, virulence, and treatment of *Candida auris*. *Pathog. Immun.* **7**(2), 46. 10.20411/pai.v7i2.535 (2022). 36329818 10.20411/pai.v7i2.535 PMC9620957

[12] Fayed, B. Nanoparticles in the battle against *Candida auris* biofilms: Current advances and future prospects. *Drug Deliv. Transl. Res.* **26**, 1–7. 10.1007/s13346-024-01749-w (2024). 10.1007/s13346-024-01749-w PMC11968567 39589626

[13] De Gaetano, S., Midiri, A., Mancuso, G., Avola, M. G. & Biondo, C. *Candida auris* outbreaks: Current status and future perspectives. *Microorganisms* **12**(5), 927. 10.3390/microorganisms12050927 (2024). 38792757 10.3390/microorganisms12050927 PMC11123812

[14] Jacobs, S. E. et al. *Candida auris* pan-drug-resistant to four classes of antifungal agents. *Antimicrob. Agents Chemother.* **66**(7), e00053-e122. 10.1128/aac.00053-22 (2022). 35770999 10.1128/aac.00053-22 PMC9295560

[15] Černáková, L., Roudbary, M., Brás, S., Tafaj, S. & Rodrigues, C. F. *Candida auris*: A quick review on identification, current treatments, and challenges. *Int. J. Mol. Sci.* **22**(9), 4470. 10.3390/ijms22094470 (2021). 33922907 10.3390/ijms22094470 PMC8123192

[16] Suphavilai, C. et al. Detection and characterisation of a sixth *Candida auris* clade in Singapore: A genomic and phenotypic study. *Lancet Microbe* **5**(9), 100878. 10.1016/S2666-5247(24)00101-0 (2024). 39008997 10.1016/S2666-5247(24)00101-0

[17] Chow, N. A. et al. Tracing the evolutionary history and global expansion of *Candida auris* using population genomic analyses. *MBio* **11**(2), 10–128. 10.1128/mBio.03364-19 (2020). 10.1128/mBio.03364-19 PMC7188998 32345637

[18] Kappel, D. et al. Genomic epidemiology describes introduction and outbreaks of antifungal drug-resistant *Candida auris*. *NPJ Antimicrob. Resist.* **2**(1), 1–10. 10.1038/s44259-024-00043-6 (2024). 39359891 10.1038/s44259-024-00043-6 PMC11442302

[19] Ahmad, S., Khan, Z., Al-Sweih, N., Alfouzan, W. & Joseph, L. *Candida auris* in various hospitals across Kuwait and their susceptibility and molecular basis of resistance to antifungal drugs. *Mycoses* **63**(1), 104–112. 10.1111/myc.13022 (2020). 31618799 10.1111/myc.13022

[20] Ahmad, S. & Asadzadeh, M. Strategies to prevent transmission of *Candida auris* in healthcare settings. *Curr. Fungal Infect. Rep.* **17**(1), 36–48. 10.1007/s12281-023-00451-7 (2023). 36718372 10.1007/s12281-023-00451-7 PMC9878498

[21] Ain Shams University Specialized Hospital. About the Hospital, accessed July 15, 2025; https://asush.asu.edu.eg/abouts.

[22] Bassetti, M. et al. EORTC/MSGERC Definitions of invasive fungal diseases: Summary of activities of the intensive care unit working group. *Clin. Infect. Dis.* **72**(2), S121–S127. 10.1093/cid/ciaa1751 (2021). 33709127 10.1093/cid/ciaa1751

[23] Centers for Disease Control and Prevention (CDC). Identification of C. auris. *CDC*. (2019).https://www.cdc.gov/candida-auris/hcp/laboratories/identification-of-c-auris.html

[24] Ambaraghassi, G. et al. Identification of *Candida auris* by use of the updated Vitek 2 yeast identification system, version 8.01: A multilaboratory evaluation study. *J. Clin. Microbiol.* **57**(11), 10–128. 10.1128/JCM.00884-19 (2019). 10.1128/JCM.00884-19 PMC6812989 31413079

[25] Centers for Disease Control and Prevention (CDC). *Detection of Colonization* (CDC, 2024). https://www.cdc.gov/candida-auris/hcp/laboratories/detection-colonization.html

[26] Abdolrasouli, A. & Fraser, M. A. Candida auris identification and profiling by MALDI–ToF mass spectrometry. In *Candida auris Methods in Molecular Biology* Vol. 2517 (ed. Lorenz, A.) 21–32 (Humana, 2022). 10.1007/978-1-0716-2417-3_2. 10.1007/978-1-0716-2417-3_2 35674942

[27] Clinical and Laboratory Standards Institute (CLSI). Reference method for broth dilution antifungal susceptibility testing of yeasts; approved standard-third edition. CLSI document M27-A3 (CLSI, 2008).

[28] Centers for Disease Control and Prevention (CDC). *Antifungal Susceptibility Testing* (CDC, 2024). https://www.cdc.gov/candida-auris/hcp/laboratories/antifungal-susceptibility-testing.html

[29] Arendrup, M. C., Prakash, A., Meletiadis, J., Sharma, C. & Chowdhary, A. Comparison of EUCAST and CLSI reference microdilution MICs of eight antifungal compounds for *candida auris* and associated tentative epidemiological cutoff values. *Antimicrob. Agents Chemother.* **61**(6), e00485-e517. 10.1128/aac.00485-17 (2017). 28416539 10.1128/AAC.00485-17 PMC5444165

[30] Bohner, F. et al. Acquired triazole resistance alters pathogenicity-associated features in *candida auris* in an isolate-dependent manner. *J. Fungi* **9**(12), 1148. 10.3390/jof9121148 (2023). 10.3390/jof9121148 PMC10744493 38132749

[31] Phan-Canh, T., Nguyen-Le, D., Luu, P., Khunweeraphong, N. & Kuchler, K. Rapid in vitro evolution of flucytosine resistance in *Candida auris*. *mSphere* **10**(3), e00977-e1024. 10.1128/msphere.00977-24 (2025). 40099908 10.1128/msphere.00977-24 PMC12039228

[32] Yenisehirli, G., Bulut, N., Yenisehirli, A. & Bulut, Y. In vitro susceptibilities of Candida albicans isolates to antifungal agents in Tokat, Turkey. *Jundishapur J. Microbiol.* **8**(9), e28057. 10.5812/jjm.28057 (2015). 26495115 10.5812/jjm.28057 PMC4609313

[33] Kroustali, V. et al. Antifungal susceptibility testing and determination of local epidemiological cut-off values for Candida species isolated from women with vulvovaginal candidiasis. *Microbiol. Spectr.* **13**(3), e02488-e2524. 10.1128/spectrum.02488-24 (2025). 39846759 10.1128/spectrum.02488-24 PMC11878056

[34] Welsh, R. M. et al. Survival, persistence, and isolation of the emerging multidrug-resistant pathogenic yeast *Candida auris* on a plastic health care surface. *J. Clin. Microbiol.* **55**(10), 2996–3005. 10.1128/JCM.00921-17 (2017). 28747370 10.1128/JCM.00921-17 PMC5625385

[35] Semenya, M. D., Aladejana, A. E. & Ndlovu, S. I. Characterization of susceptibility patterns and adaptability of the newly emerged *Candida auris*. *Int. Microbiol.* **28**, 575–587. 10.1007/s10123-024-00563-1 (2025). 39107585 10.1007/s10123-024-00563-1 PMC11906518

[36] Oyardi, O. et al. Phenotypic investigation of virulence factors, susceptibility to Ceragenins, and the impact of biofilm formation on drug efficacy in *Candida auris* isolates from Türkiye. *J. Fungi* **9**(10), 1026. 10.3390/jof9101026 (2023). 10.3390/jof9101026 PMC10607835 37888282

[37] Bansal, M., Samant, S. A., Singht, S. & Talukdar, A. Phenotypic detection of biofilms in Candida species isolated from various clinical samples. *Int. J. Curr. Microbiol. App. Sci.* **5**(3), 47–56. 10.20546/ijcmas.2016.503.007 (2016).

[38] Dhanasekaran, D., Vinothini, K., Latha, S., Thajuddin, N. & Panneerselvam, A. Human dental biofilm: Screening, characterization, in vitro biofilm formation and antifungal resistance of Candida spp. *Saudi J. Dent. Res.* **5**(1), 55–70. 10.1016/j.ksujds.2013.10.001 (2014).

[39] Chowdhary, A., Tarai, B., Singh, A. & Sharma, A. Multidrug-resistant *Candida auris* infections in critically ill coronavirus disease patients, India, April-July 2020. *Emerg Infect Dis.* **26**(11), 2694. 10.3201/eid2611.203504 (2020). 32852265 10.3201/eid2611.203504 PMC7588547

[40] Eix, E. F. & Nett, J. E. *Candida auris*: Epidemiology and antifungal strategy. *Annu. Rev. Med.* **76**(1), 57. 10.1146/annurev-med-061523-021233 (2024). 10.1146/annurev-med-061523-021233 PMC11808652 39656947

[41] Osman, M. et al. Update on invasive fungal infections in the Middle Eastern and North African region. *Braz. J. Microbiol.* **51**(4), 1771–1789. 10.1007/s42770-020-00325-x (2020). 32623654 10.1007/s42770-020-00325-x PMC7335363

[42] Osaigbovo, I. et al. *Candida auris*: A systematic review of a globally emerging fungal pathogen in Africa. *Open Forum Infect. Dis.* **11**(6), ofad681. 10.1093/ofid/ofad681 (2023). 38887473 10.1093/ofid/ofad681 PMC11181182

[43] El-Kholy, M., Shawky, S., Fayed, A. & Meis, J. *Candida auris* bloodstream infection in Egypt. In: 9th trends in medical mycology held on 11–14 October 2019, Nice, France, Organized under the Auspices of EORTC-IDG and ECMM. *J Fungi. ****5***(4), 310–1 (2019). 10.3390/jof5040095 10.3390/jof5040095 PMC7807382 31597285

[44] Khairat, S. M., Anany, M. G., Ashmawy, M. M. & Hussein, A. F. Setting a protocol for identification and detecting the prevalence of *Candida auris* in tertiary Egyptian hospitals using the CDC steps. *Open Access Maced J. Med. Sci.* **9**(A), 397–402. 10.3889/oamjms.2021.6095 (2021).

[45] Mahmoud, N., Mohamed, M. A., Hafez, R. M. & Moussa, T. A. Bloodstream infection with *Candida auris* in Egypt. *Biosci. Res.* **20**(4), 1–13 (2024).

[46] Spruijtenburg, B. et al. Genetic epidemiology and resistance investigations of clinical yeasts in Alexandria, Egypt. *Pathogens* **14**(5), 486. 10.3390/pathogens14050486 (2025). 40430806 10.3390/pathogens14050486 PMC12114656

[47] Centers for Disease Control and Prevention (CDC). *Candida auris* testing algorithm by method. *CDC*, accessed 17 July 2025; https://www.cdc.gov/candida-auris/media/pdfs/Testing-algorithm_by-Method_508.pdf (2019).

[48] Giacobbe, D. R. et al. Challenges in the diagnosis and treatment of candidemia due to multidrug-resistant *Candida auris*. *Front. Fungal Biol.* **4**, 1061150. 10.3389/ffunb.2023.1061150 (2023). 37746122 10.3389/ffunb.2023.1061150 PMC10512377

[49] Fasciana, T. et al. *Candida auris*: An overview of how to screen, detect, test and control this emerging pathogen. *Antibiotics* **9**(11), 778. 10.3390/antibiotics9110778 (2020). 33167419 10.3390/antibiotics9110778 PMC7694398

[50] Alfouzan, W. et al. Molecular epidemiology of *Candida auris* outbreak in a major secondary-care hospital in Kuwait. *J. Fungi* **6**(4), 307. 10.3390/jof6040307 (2020). 10.3390/jof6040307 PMC7712429 33233388

[51] Du, H. et al. *Candida auris*: Epidemiology, biology, antifungal resistance, and virulence. *PLoS Pathog.* **16**(10), e1008921. 10.1371/journal.ppat.1008921 (2020). 33091071 10.1371/journal.ppat.1008921 PMC7581363

[52] Hayes, J. F. *Candida auris*: Epidemiology update and a review of strategies to prevent spread. *J. Clin. Med.* **13**(22), 6675. 10.3390/jcm13226675 (2024). 39597821 10.3390/jcm13226675 PMC11595167

[53] Scolarici, M., Jorgenson, M., Saddler, C. & Smith, J. Fungal infections in liver transplant recipients. *J. Fungi* **7**(7), 524. 10.3390/jof7070524 (2021). 10.3390/jof7070524 PMC8304186 34210106

[54] Vu, C. A., Jimenez, A., Anjan, S. & Abbo, L. M. Challenges and opportunities in stewardship among solid organ transplant recipients with *Candida auris* bloodstream infections. *Transpl. Infect. Dis.* **24**(5), e13919. 10.1111/tid.13919 (2022). 36254515 10.1111/tid.13919

[55] Deshkar, S. et al. Identification and antifungal drug susceptibility pattern of *Candida auris* in India. *J. Glob. Infect. Dis.* **14**(4), 131–135. 10.4103/jgid.jgid_44_22 (2022). 36636301 10.4103/jgid.jgid_44_22 PMC9831210

[56] Dickwella Widanage, M. C. et al. Distinct echinocandin responses of Candida albicans and *Candida auris* cell walls revealed by solid-state NMR. *Nat. Commun.* **16**(1), 1–17. 10.1038/s41467-025-61678-1 (2025). 40628778 10.1038/s41467-025-61678-1 PMC12238410

[57] Ahmed, S. H., El-Kholy, I. M., El-Mehalawy, A. A., Mahmoud, E. M. & Elkady, N. A. Molecular characterization of some multidrug resistant *Candida auris* in Egypt. *Sci. Rep.* **15**(1), 4917. 10.1038/s41598-025-88656-3 (2025). 39929931 10.1038/s41598-025-88656-3 PMC11811120

[58] Siopi, M. et al. Evaluation of the Vitek 2 system for antifungal susceptibility testing of *Candida auris* using a representative international panel of clinical isolates: Overestimation of amphotericin B resistance and underestimation of fluconazole resistance. *J. Clin. Microbiol.* **62**(4), e01528-e1623. 10.1128/jcm.01528-23 (2024). 38501836 10.1128/jcm.01528-23 PMC11005389

[59] Alonso-Monge, R., Cortés-Prieto, I., Román, E. & Pla, J. Morphogenetic transitions in the adaptation of Candida albicans to the mammalian gut. *Microbes Infect.* **26**(3), 105253. 10.1016/j.micinf.2023.105253 (2024). 37977323 10.1016/j.micinf.2023.105253

[60] Hernando-Ortiz, A. et al. Virulence of *Candida auris* from different clinical origins in Caenorhabditis elegans and Galleria mellonella host models. *Virulence.* **12**(1), 1063–1075. 10.1080/21505594.2021.1908765 (2021). 33843456 10.1080/21505594.2021.1908765 PMC8043173

[61] ElBaradei, A. A decade after the emergence of *Candida auris*: What do we know?. *Eur. J. Clin. Microbiol. Infect. Dis.* **39**(9), 1617–1627. 10.1007/s10096-020-03886-9 (2020). 32297040 10.1007/s10096-020-03886-9

[62] Rossato, L. & Colombo, A. L. *Candida auris*: What have we learned about its mechanisms of pathogenicity?. *Front. Microbiol.* **9**, 3081. 10.3389/fmicb.2018.03081 (2018). 30631313 10.3389/fmicb.2018.03081 PMC6315175

[63] Bravo Ruiz, G., Ross, Z. K., Gow, N. A. & Lorenz, A. Pseudohyphal growth of the emerging pathogen *Candida auris* is triggered by genotoxic stress through the S phase checkpoint. *mSphere.* **5**(2), e00151-e220. 10.1128/msphere.00151-20 (2020). 32161147 10.1128/mSphere.00151-20 PMC7067593

[64] Osei Sekyere, J. *Candida auris*: A systematic review and meta-analysis of current updates on an emerging multidrug-resistant pathogen. *Microbiologyopen* **7**(4), e00578. 10.1002/mbo3.578 (2018). 29345117 10.1002/mbo3.578 PMC6079168

[65] Chybowska, A. D., Childers, D. S. & Farrer, R. A. Nine things genomics can tell us about *Candida auris*. *Front. Genet.* **11**, 351. 10.3389/fgene.2020.00351 (2020). 32351544 10.3389/fgene.2020.00351 PMC7174702

[66] Carvajal, S. K. et al. Pathogenicity assessment of Colombian strains of *Candida auris* in the Galleria mellonella invertebrate model. *J. fungi* **7**(6), 401. 10.3390/jof7060401 (2021). 10.3390/jof7060401 PMC8223996 34063862

[67] Kim, J. S., Cha, H. & Bahn, Y. S. Comprehensive overview of *Candida auris*: An emerging multidrug-resistant fungal pathogen. *J. Microbiol. Biotechnol.* **34**(7), 1365–1375. 10.4014/jmb.2404.04040 (2024). 38881183 10.4014/jmb.2404.04040 PMC11294645

[68] Mroczyńska, M. & Brillowska-Dąbrowska, A. Virulence of clinical Candida isolates. *Pathogens* **10**(4), 466. 10.3390/pathogens10040466 (2021). 33921490 10.3390/pathogens10040466 PMC8070227

[69] Nouraei, H., Jahromi, M. G., Jahromi, L. R., Zomorodian, K. & Pakshir, K. Potential pathogenicity of Candida species isolated from oral cavity of patients with diabetes mellitus. *Biomed. Res. Int.* **2021**, 9982744. 10.1155/2021/9982744 (2021). 34136578 10.1155/2021/9982744 PMC8175137

[70] Wan, L. et al. Changes in the hemolytic activity of Candida species by common electrolytes. *BMC microbiol.* **15**, 171. 10.1186/s12866-015-0504-7 (2015). 26296996 10.1186/s12866-015-0504-7 PMC4546287

[71] Inci, M. et al. Investigating virulence factors of clinical Candida isolates in relation to atmospheric conditions and genotype. *Turk. J. Med. Sci.* **42**(8), 1476–1483 (2012).

[72] Yigit, N. & Aktas, E. Comparison of efficacy of different blood medium in determining hemolytic activity of Candida species. *Eurasian J. Med.* **41**(2), 95 (2009). 25610076 PMC4299835

[73] Singh, R., Kaur, M., Chakrabarti, A., Shankarnarayan, S. A. & Rudramurthy, S. M. Biofilm formation by *Candida auris* isolated from colonising sites and candidemia cases. *Mycoses* **62**(8), 706–709. 10.1111/myc.12947 (2019). 31132181 10.1111/myc.12947

[74] Bing, J. et al. Clinical isolates of *Candida auris* with enhanced adherence and biofilm formation due to genomic amplification of ALS4. *PLoS Path.* **19**(3), e1011239. 10.1371/journal.ppat.1011239 (2023). 10.1371/journal.ppat.1011239 PMC10035925 36913408

[75] Louvet, M. et al. Ume6-dependent pathways of morphogenesis and biofilm formation in *Candida auris*. *Microbiol. Spectr.* **12**(11), e01531. 10.1128/spectrum.01531-24 (2024). 39297645 10.1128/spectrum.01531-24 PMC11537075

